# Radioimmunotherapy of Blastomycosis in a Mouse Model With a (1→3)-β-Glucans Targeting Antibody

**DOI:** 10.3389/fmicb.2020.00147

**Published:** 2020-02-07

**Authors:** Muath Helal, Kevin J. H. Allen, Bruce van Dijk, Joshua D. Nosanchuk, Elisabeth Snead, Ekaterina Dadachova

**Affiliations:** ^1^College of Pharmacy and Nutrition, University of Saskatchewan, Saskatoon, SK, Canada; ^2^Department of Orthopedics, University Medical Center Utrecht, Utrecht, Netherlands; ^3^Department of Medicine, Albert Einstein College of Medicine, The Bronx, NY, United States; ^4^Western College of Veterinary Medicine, University of Saskatchewan, Saskatoon, SK, Canada

**Keywords:** radioimmunotherapy, *Blastomyces dermatitidis*, (1→3)-β-glucan, mouse model, 213Bismuth

## Abstract

Invasive fungal infections (IFI) cause devastating morbidity and mortality, with the number of IFIs more than tripling since 1979. Our laboratories were the first to demonstrate that radiolabeled microorganism-specific monoclonal antibodies are highly effective for treatment of experimental fungal, bacterial and viral infections. Later we proposed to utilize surface expressed pan-antigens shared by major IFI-causing pathogens such as beta-glucans as RIT targets. Here we evaluated *in vivo* RIT targeting beta-glucan in *Blastomyces dermatitidis* which causes serious infections in immunocompromised and immunocompetent individuals and in companion dogs. *B. dermatitidis* cells were treated with the 400-2 antibody to (1→3)-β-glucans radiolabeled with the beta-emitter 177Lutetium (^177^Lu) and alpha-emitter 213Bismuth (^213^Bi) and the efficacy of cell kill was determined by colony forming units (CFUs). To determine the antigen-specific localization of the 400-2 antibody *in vivo*, C57BL6 mice were infected intratracheally with 2 × 10^5^ *B. dermatitidis* cells and given ^111^In-400-2 antibody 24 h later. To evaluate the killing of *B. dermatitidis* cells with RIT, intratracheally infected mice were treated with 150 μCi ^213^Bi-400-2 and their lungs analyzed for CFUs 96 h post-infection. ^213^Bi-400-2 proved to be more effective in killing *B. dermatitidis* cells *in vitro* than ^177^Lu-400-2. Three times more ^111^In-400-2 accumulated in the lungs of infected mice, than in the non-infected ones. ^213^Bi-400-2 lowered the fungal burden in the lungs of infected mice more than 2 logs in comparison with non-treated infected controls. In conclusion, our results demonstrate the ability of an anti-(1-3)-beta-D-glucan antibody armed with an alpha-emitter ^213^Bi to selectively kill *B. dermatitidis* cells *in vitro* and *in vivo*. These first *in vivo* results of the effectiveness of RIT targeting pan-antigens on fungal pathogens warrant further investigation.

## Introduction

Invasive fungal infections (IFI) cause devastating morbidity and mortality, especially in organ transplant patients, cancer patients and patients in intensive care units, with the number of IFIs more than tripling since 1979 (Enoch et al., 2017). There are three main categories of medications used for the treatment of IFI: polyenes (primarily amphotericin B), azoles (fluconazole, itraconazole, voriconazole, posaconazole), and echinocandins (caspofungin, micafungin, anidulafungin). Additionally, flucytosine is primarily utilized in combination with amphotericin B for the treatment of cryptococcosis. Hence, the number of medications available to combat mycostic infections is significantly less than what is available for treating bacterial diseases and the number of targets of these antifungal medications are far fewer (polyenes and azoles: cell membrane sterols; echinocandins: cell wall beta-1,3-glucans; flucytosine: RNA, DNA). Furthermore, the echinocandins, the last new class approved by the Food and Drug Administration (FDA), have been available for ∼10 years with no appearance of new drug classes for the severe fungal diseases.

Radioimmunotherapy (RIT) utilizes antigen-antibody interaction to deliver lethal doses of ionizing radiation to cells and has demonstrated efficacy in several types of cancer (Tomblyn, 2012; Larson et al., 2015). The distinct advantages of RIT over other drugs are: (1) its cytocidal nature, meaning that RIT does not merely abrogate a single cellular pathway but physically destroys targeted cells or cellular machinery; (2) it is less subject to drug resistance mechanisms; (3) its efficacy is independent of the immune status of a host; and (4) it has low toxicity in comparison to chemotherapy due to the specific tumor targeting.

Our laboratories were the first to demonstrate that microorganism-specific monoclonal antibodies (mAbs) are highly effective for the treatment of experimental fungal, bacterial and viral infections (Dadachova et al., 2003; reviewed in Helal and Dadachova, 2018) as well as virally induced cancers (Wang et al., 2007; Phaeton et al., 2016). Using *Cryptococcus neoformans* as a model organism and the antibodies to its polysaccharide capsule as targeting molecules for the radionuclides we investigated various aspects of RIT of fungal disease such as long term efficacy of the treatment with infected mice being observed for up to 75 days post RIT, high inoculum, acute versus established infection (Dadachova et al., 2003; Bryan et al., 2009; Jiang et al., 2012). We also investigated the radiobiological and immunological mechanisms of actions of infections RIT (Dadachova et al., 2006a, b; Bryan et al., 2008). Several years ago, we proposed that we can utilize surface-expressed antigens shared by major IFI-causing pathogens as targets for radiolabeled mAbs (Nosanchuk and Dadachova, 2012). This approach is different than that utilized to date in cancer RIT, where the selected mAbs target a specific cell type. Fortunately, IFI-causing fungi do share common cell wall associated antigens that also constitute major virulence factors for these fungi, which we called “pan-antigens,” such as melanin, heat shock protein 60 (HSP60) (Guimarães et al., 2009) and beta-glucans. These antigens are exposed on the surface of fungal cells and thus are accessible to the radiolabeled mAbs for binding and delivering cytocidal payloads to those cells. Using *C. neoformans* and *Candida albicans* as model organisms, our *in vitro* experiments showed that antibodies to the above pan-antigens killed 80–100% of fungal cells when radiolabeled with alpha-particles emitting radionuclide 213Bismuth (Bryan et al., 2012).

In this work, we provide first experimental *in vivo* evidence that pan-antigens on IFI causing pathogens can be targeted with RIT. *Blastomycosis dermatitidis* was chosen as a model for this project. It is an example of an invasive fungal pathogen that causes serious infections in immunocompromised patients (Pappas et al., 1993), immunocompetent individuals (Gray and Baddour, 2002) and in companion dogs (Davies et al., 2013). It has relatively high prevalence in different areas in Canada and the United States (Davies et al., 2013) and the endemic regions may be increasing, as evidenced by reports in New York, Vermont, Texas, Nebraska, and Kansas (McDonald et al., 2018). We have chosen (1→3)-β-glucan as an RIT target because of the encouraging results in our *in vitro* work (Bryan et al., 2012) and because the antibodies to this antigen are commercially available. We have demonstrated that those antibodies in the radiolabeled form bind specifically to *B. dermatitidis* cells *in vitro* and *in vivo* and that their administration to infected mice results in several logs reduction of the infectious burden.

## Materials and Methods

### Fungal Cultures and Antibodies

Acapsular *C. neoformans* (ATCC 208821) cells were cultured in Emmons’ modification of Sabouraud’s agar overnight (37°C) until sufficient cell numbers were reached. Wild type strain of *B. dermatitidis* Gilchrist et Stokes (ATCC 26199) was used in all experiments. As 26199 strain is a Biological Safety Level (BSL) 3 pathogen in Canada, all manipulations with the cells were conducted using standard operating procedures which enhanced BSL2 (BSL2 +) facilities with the approval by the Public Health Agency of Canada and by the Biosafety Office at University of Saskatchewan. To prevent the formation of the potentially infectious spores, the cells were kept at 37°C at all times except when frozen for storage. *B. dermatitidis* cells were cultured in *Histoplasma* macrophage medium (HMM) (Worsham and Goldman, 1988), at 37°C for 5–7 days until sufficient numbers of cells were reached. The media was prepared by dissolving glucose (18.2 g), glutamic acid (1.0 g), cysteine (1.0 g) and HEPES (6.0 g) in 1 L of water. The solution was filter sterilized followed by the addition of 10.6 g of F-12 Nutrient mixture (cat. number: N6760, Sigma). Passage 2–4 were used throughout the work. Agar was prepared by suspending 19.47 g of MiddleBrook 7H10 Agar Base (cat. number: M0303, Sigma) in 900 ml distilled water followed by the addition of 5 ml glycerol. The mixture was then autoclaved and left at room temperature to cool down. Dubos Oleic Albumin Complex (cat. number: 215333, BD Difco) was added as a media additive to enhance the growth of *B. dermatitidis.* The agar was poured in Petri dishes and left to solidify. The dishes were left at 4°C until needed. Murine IgG2b mAb 2G8 to (1→3)-β-glucan (Torosantucci et al., 2005) was produced by Diatheva Srl, Cartoceto, Italy, and was a kind gift from Dr. A. Torosantucci (Istituto Superiore di Sanità, Rome, Italy). Its Kd is 1.34 × 10^–9^ M according to the manufacturer’s data. 400-2 murine IgG1 mAb to (1→3)-β-glucan was procured from Biosupplies Australia Pty. Ltd. (Parkville, Australia). The murine isotype control IgG1 antibody MOPC-21 was procured from Invitrogen (Waltham, United States).

### Radionuclides and Radiolabeling of Antibodies

400-2, 2G8 and MOPC21 antibodies were first conjugated to a chelating agent *C*-functionalized *trans*-cyclohexyldiethylene-triamine pentaacetic acid derivative (CHXA″) (Macrocyclics, San Antonio, TX, United States) with a 10 molar excess to enable its subsequent radiolabeling with ^111^In, ^177^Lu or ^213^Bi. Conjugation and labeling was carried out according to a previously published method (Allen et al., 2018, 2019). In short, the conjugation reaction was carried out for 1.5 h at 37°C in sodium carbonate buffer at pH 8.5. The reaction was followed by exchanging the buffer into 0.15 M ammonium acetate buffer, pH 6.5. ^225^Ac for construction of the ^213^Bi/^225^Ac radionuclide generator was purchased from Oak Ridge National Laboratory, TN, United States. ^177^Lu in form of ^177^Lu chloride was acquired from RadioMedix (TX, United States), and ^111^In chloride – from BWXT Canada. The CHXA″- conjugated antibodies were radiolabeled with ^111^In, ^177^Lu and ^213^Bi. The radiolabeling of CHXA″ conjugates with ^111^In and ^177^Lu was performed to achieve the specific activity of 5 μCi/μg of the antibody. 800 μCi of ^111^In or ^177^Lu chloride was added to 10 μL 0.15 M ammonium acetate buffer and added to a microcentrifuge tube containing 160 μ*g* of the desired CHXA″ conjugated antibody in 0.15 M ammonium acetate buffer. The reaction mixture was incubated for 60 min at 37°C, and then the reaction was quenched by the addition of 3 μL of 0.05 M EDTA solution. For radiolabeling with ^213^Bi, it was eluted from a ^213^Bi/^225^Ac radionuclide generator with a 300 μL 0.1 M hydroiodic acid (HI) solution followed by 300 μL milliQ H_2_O. The pH of the solution was adjusted to 6.5 with 80 μL of 5 M ammonium acetate buffer prior to addition to the desired CHXA″ conjugated antibody and the reaction mixture was incubated for 5 min at 37°C. The ^213^Bi-labeled mAbs were purified from HI on a 0.5 mL Amicon disposable size exclusion filter (30 K MW cut off, Fisher). The percentage of radiolabeling was measured by QQsilica gel-instant thin layer chromatography (SG-iTLC) using 0.15 M ammonium acetate buffer as the eluent (top containing unlabeled ^111^In/^213^Bi/^177^L, bottom containing radiolabeled antibody). SG-iTLCs were read on a PerkinElmer 2470 Automatic Gamma Counter. Greater than 98% labeling was routinely achieved and ^111^In- and ^177^Lu-labeled mAbs were used immediately with no need for further purification.

### Determination of the Binding Ability of Radiolabeled Antibodies to Fungal Cell Surfaces

We radiolabeled fungal beta-glucan-specific (400-2 or 2G8) mAbs with ^177^Lu. Five million acapsular *C. neoformans* cells were incubated for 3 h (37°C) with a radiolabeled mAb in albumin-pre-coated Eppendorf tubes. A radiolabeled isotype antibody (^177^Lu-MOPC-21) was also used as a control. In addition, samples with radiolabeled mAb without cells were used to rule out non-specific binding to the plastic tubes. Radiolabeled antibody at a specific activity of 5:1 (25 μCi or 50 μCi radioactivity) were used in this experiment. Following incubation, the tube with the cells and the radiolabeled antibody was measured using PerkinElmer© Gamma Counter. The cells were then centrifuged, the supernatant was removed and its radioactivity measured in a gamma counter. The cellular pellet was washed twice with phosphate buffered saline (PBS), and the radioactivity of the pellet was measured in a gamma counter. The percentages of the radiolabeled antibody bound to the cells and of the unbound antibody were calculated using the following formulae:

Radiolabeledantibodyboundtothepellet,%=(Counts⁢in⁢the⁢pellet/Counts⁢in⁢the⁢tube)×100

Unboundradiolabeledantibody,%=100-(Counts⁢in⁢the⁢pellet/Counts⁢in⁢the⁢tube)×100

### Determination of RIT Cytotoxicity Against *B. dermatitidis*

10^4^ *B. dermatitidis* cells were incubated with ^213^B-400-2 mAb (1 or 5 μCi at a specific activity of 5 μCi/μg) or ^177^Lu 400-2 (20 μCi at a the same specific activity) for 1 h. MOPC-21 was used as an isotype control. After 1 h incubation, the cells were centrifuged and washed to remove any radioactivity. Cells were then cultured on MiddleBrook agar at 37°C for 3 days 37°C after which the colony forming units (CFUs) were counted in each group and the percentages of cell survival were calculated.

### Biodistribution of the Radiolabeled 400-2 Antibody in *B. dermatitidis* Infected and Healthy Mice

All animal studies were approved by the Animal Research Ethics Board of the University of Saskatchewan. The objective of this experiment was to determine if the ^111^In-400-2 mAb accumulated in the *B. dermatitidis* infected lungs in mice to a higher extent than in non-infected animals. Twenty female black C57B16 mice (4–6 weeks old) were randomized into 4 groups (each with 5 mice). Only groups one and two were infected intratracheally with 2 × 10^5^ *B. dermatitidis* cells while groups three and four received PBS alone Mice received isoflurane with oxygen for anesthesia purposes to enable the intratracheal infection. After 24 h, all mice received intraperitoneal injection of ^111^In-400-2 antibody (30 μCi). Groups one and three were euthanized at 48 h post-infection while groups two and four were euthanized at 96 h post-infection. Lungs were then isolated. Radioactivity was then measured for each lung tissue using PerkinElmer© Gamma Counter in reference to the radiolabeled antibody standard as described (Allen et al., 2018). Radioactivity was then determined per unit weight of each isolated lung.

### Radioimmunotherapy of *B. dermatitidis*-Infected Mice

This experiment was conducted to measure the *in vivo* effectiveness of RIT in treating mice infected with *B. dermatitidis*. For this, 27 female black C57B16 mice were infected intratracheally with 10^6^ *B*. *dermatitidis* cells. Twenty four hours after the infection, mice were randomized into 4 groups. The first (9 mice) and second (5 mice) groups received 150 μCi ^213^Bi-400-2 and 150 μCi ^213^Bi-MOPC-21, respectively. The third group (8 mice) received no treatment, while the fourth group (5 mice) received only cold 400-2 antibody (30 μg). All treatments were given intraperitoneally. A fifth group consisted of two healthy mice which received neither infection or treatment. Mice were observed for 72 h post treatment and then euthanized. Lungs were isolated and passed through cell strainers. Aseptic conditions were maintained through all the procedures. The cells were centrifuged, concentrated in 700 μL PBS and plated on MiddleBrook agar for 24 h at 37°C. ImageJ software was then used to calculate the area on the colonies formed on the Petri dish in each sample. For the same dish, the average areas of 10 random colonies was also determined. Total area was divided by the average colony area to estimate the number of colonies formed for each plate. In addition, the aliquots of homogenized lungs were obtained from four mice in untreated infected control and ^213^Bi-400-2 groups. DNA was then isolated from the samples using GenomicPrep Mini Spin kit (Cat. Number 28-9042-58) according to the manufacturer protocol. The levels of *BAD1* and *C57BL/6L* genes were quantified in each sample using real-time PCR system (Bio-Rad CFX96 Real Time System) and SYBR Master Mix. Levels of *BAD1* and C57BL/6L correlate to those of *B. dermatitidis* and mouse cells, respectively. Primers for *BAD1* were forward (5′-AAGTGGCTGGGTAGTTATACGCTAC-3′) and reverse (5′-TAGGTTGCTGATTCCATAAGTCAGG-3′ primers) (Sidamonidze et al., 2012) while a genomic marker found in C57BL/6J mice was detected using the forward (5′-AACTCTCAGGGGTCCTGTGT-3′ and reverse (5′-CCTGGGC CTCACTTATTGGG-3′) primers. The quantity of *BAD1* was then referenced to that of C57BL/6L genomic marker in both control and ^213^Bi-400-2 groups.

### Statistical Analysis

The data are presented as mean ± standard deviation (SD). One-way ANOVA followed by Tukey’s *post hoc* test was employed to determine the significant difference between the different groups using GraphPad Prism 5 software (San Diego, CA, United States). A *p*-value less than 0.05 was considered significant.

## Results

### The Antibodies to (1→3)-β-Glucan Preserved Their Immunoreactivity After Radiolabeling

**FIGURE 1:**
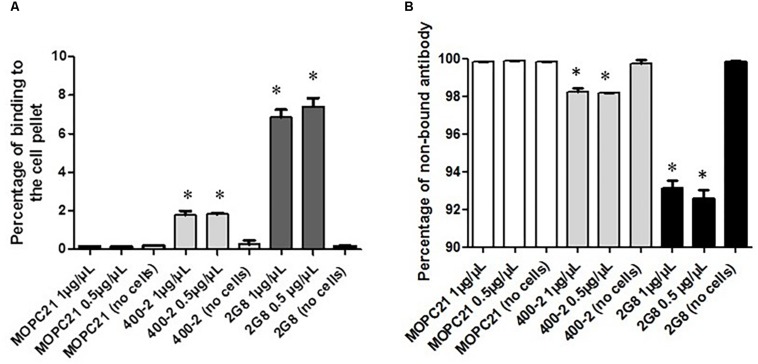
Evaluation of the binding of ^177^Lu-400-2 and ^177^Lu-2G8 antibodies to (1→3)-β-glucans antigen after their incubation with acapsular *C. neoformans* cells for 3 h. ^177^Lu-MOPC 21 was used as an IgG irrelevant control. **(A)** percentage of radiolabeled antibody in a sample bound to the cell pellet: **(B)** percentage of non-bound antibody. ^∗^Indicates a significant difference between the groups (*P* value < 0.05).

Initially, the immunoreactivity toward (1→3)-β-glucan antigen of 400-2 and 2G8 mAbs radiolabeled with ^111^In was determined by binding to acapsular *C. neoformans* cells. Results show that both 400-2 and 2G8 antibodies were able to bind significantly higher than the MOPC-21 irrelevant control mAb (Figure 1A) while there was no binding of 400-2 and 2G8 antibodies to the plates alone without the cells. Conversely, there was significantly more non-bound antibody in case of MOPC-21 than 400-2 or 2G8 (Figure 1B). There were approximately 4 × 10^13^ antibody molecules in 10 μg of the antibody added to 5 × 10^6^ cryptococcal cells. If to assume that every cell expresses around 10^4^ antigen binding sites, it means that around 16 and 50 antibody molecules were bound to every cryptococcal cell for 400-2 and 2G8 mAbs, respectively. As Kd for 2G8 is 1.34 × 10^–9^ M according to the manufacturer, Kd for 400-2 was estimated from the binding data to be 4 × 10^–9^ M. As commercial availability of 2G8 antibody for further work was limited, 400-2 mAb was utilized in all subsequent experiments.

### Radiolabeled mAb 400-2 Specifically Killed *B. dermatitidis* Cells *in vitro*

**FIGURE 2:**
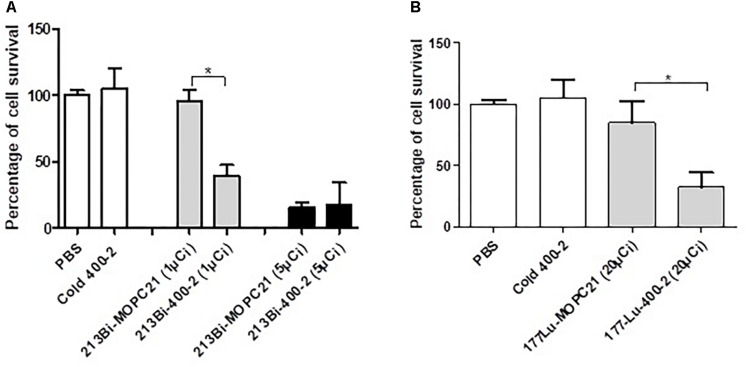
Cytotoxicity of radioimmunotherapy (RIT) toward *B. dermatitidis.* **(A)** ^213^Bi-400-2 mAb; **(B)** ^177^Lu-400-2 mAb. Cells were incubated with the radiolabeled antibodies for 1 h at 37°C followed by plating for determination of CFUs. Samples treated with PBS, cold 400-2 mAb and ^213^Bi- or ^177^Lu–labeled MOPC-21 mAb were used as controls. *Indicates a significant difference between the groups (*P* value < 0.05).

*In vitro* results show a significantly higher reduction in cell survival when treated with an alpha-emitter labeled ^213^Bi-400-2 (1 μCi) antibody compared to ^213^Bi-MOPC-21 (1 μCi), cold 400-2 and PBS controls (Figure 2A). No significant difference was observed between ^213^Bi-400-2 and ^213^Bi-MOPC-21 treated cells when the radioactivity was 5 μCi, which can be explained by non-specific killing due to the high radioactivity concentration in a small volume. When a beta-emitter ^177^Lu was used for radiolabeling, ^177^Lu-400-2 mAb also demonstrated significant cell killing in comparison to ^177^Lu-MOPC-21, cold 400-2 and PBS controls (Figure 2B).

### Biodistribution of Radiolabeled mAb 400-2 in Infected and Healthy Mice Showed Preferential Uptake in the Infected Lungs

**FIGURE 3:**
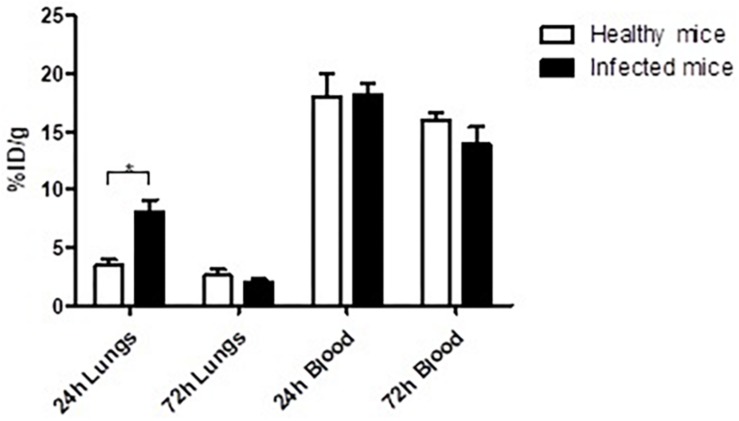
Biodistribution of ^111^In-400-2 mAb in *B. dermatitidis*-infected and in healthy mice. C57Bl6 mice were infected intratracheally with *B. dermatitidis* 2 × 10^5^ cells, injected IP with ^111^In-400-2 mAb 24 h after infection, and euthanized at 24 or 72 h post antibody administration. Five animals per group were used. %ID/g represents the percentage of injected dose of the radiolabeled antibody per gram tissue (blood and lungs). *Indicates a significant difference between the groups (*P* value < 0.05).

The biodistribution of ^111^In-400-2 in *B. dermatitidis*-infected and healthy mice was performed to evaluate the ability of 400-2 mAb to bind to *B. dermatitidis* in the lungs of infected mice. Our results showed significantly higher accumulation of ^111^In-400-2 in the lungs of infected mice compared to non-infected ones at 24 h post-injection of the antibody (Figure 3) with the uptake equalizing between infected and non-infected lungs at 72 h after antibody administration. There was no significant difference between injected dose per gram in the blood of infected and non-infected mice at both the 24 and 72 h time points (Figure 3).

### RIT of *B. dermatitidis*-Infected Mice Reduced the Fungal Burden in the Lungs by Several Logs

**FIGURE 4:**
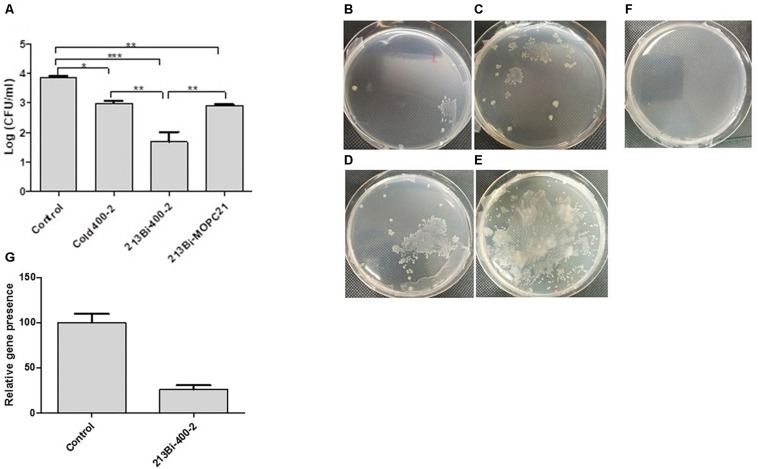
Radioimmunotherapy (RIT) of *B. dermatitidis* infected mice. **(A)** CFUs in the lungs of the mice infected with 10^6^ *B. dermatitidis* cells for 24 h and then either left untreated (control) or treated intraperitoneally with 30 μg 400-2 cold antibody, 150 μCi ^213^Bi-400-2, or 150 μCi ^213^Bi-MOPC-21 and were euthanized at 96 h post-infection. * Indicates *P* value < 0.05; ** < 0.01; *** < 0.001. **(B–F)** images of representative plates seeded with the homogenates from lungs isolated from various treatment groups: **(B)** ^213^Bi-400-2; **(C)** ^213^Bi-MOPC-21; 1 **(D)** 400-2 cold antibody; **(E)** no treatment; **(F)** uninfected, untreated mice; **(G)** real time PCR results, showing *B. dermatitidis BAD1* gene levels significantly (*p* = 0.001) lowered in the lungs of mice that received ^213^Bi-400-2 treatment in comparison with untreated infected controls.

This experiment was conducted to determine the ability of ^213^Bi-labeled 400-2 mAb to reduce the burden of infection in *B. dermatitidis-*infected mice. Results demonstrated that single administration of ^213^Bi-400-2 mAb resulted in more than 2 logs reduction in CFUs in comparison with untreated controls and in more than 1 log reduction – in comparison with the unlabeled (“cold”) and ^213^Bi-MOPC-21 groups (Figures 4A–F). The cold and ^213^Bi-MOPC-21 antibodies treatments reduced the CFUs by 1 log, this is probably attributable to opsonisation and non-specific radiation killing of fungal cells, respectively. However, the decrease in fungal burden in control groups was significantly (*p* < 0.05) less than what was observed in the ^213^Bi-400-2 group. The PCR results show that *B. dermatitidis BAD1* gene levels were significantly (*p* = 0.001) lowered in the lungs of mice that received ^213^Bi-400-2 treatment in comparison with the untreated infected controls (Figure 4G).

## Discussion

Our laboratories are developing RIT targeting pan-antigens found in all major human pathogenic fungi as a novel approach for the treatment of IFI (Bryan et al., 2012; Nosanchuk and Dadachova, 2012). This approach is a potential alternative to treat invasive infections that are difficult to cure with currently available drugs. In this study, we employed a radiolabeled antibody (^213^Bi-400-2) that targets fungal (1-3)-beta-D-glucan antigen for delivery of a potent radionuclide as a novel and potent treatment option to treat experimental blastomycosis in mice. Additionally, as (1-3)-beta-D-glucan is expressed in a wide range of fungal cells (Odabasi et al., 2006), we anticipate that radiolabeled antibodies to this antigen could be effective in treating a wide range of fungal diseases.

Before proceeding with *in vivo* experiments, we evaluated the specificity of 400-2 antibody binding to *B. dermatitidis* cells *in vitro* which was done in the absence of mammalian cells (Figure 1). 400-2 antibody to beta-glucan demonstrated binding to *B. dermatitidis* cells while the control antibody MOPC-21 – did not. These results provided reassurance that 400-2 is binding specifically to *B. dermatitidis*. We also compared the *in vitro* killing ability toward *B. dermatitidis* cells of 400-2 mAb when conjugated to two different radionuclides, an alpha emitter ^213^Bi and a beta emitter ^177^Lu. Although both radioisotopes were able to kill significant percentage of *B. dermatitidis* cells, ^213^Bi showed a higher potency than ^177^Lu. That is, 20 μCi ^177^Lu-400-2 was required to cause cell death equivalent to the damage caused by 1 μCi ^213^Bi-400-2. Interestingly, fungal cytotoxicity of radiolabeled 400-2 was observed despite the relatively low expression levels of (1-3)-beta-D-glucan in these cells (Girouard et al., 2007). These results are concordant with our previous results on *in vitro* killing with ^213^Bi-2G8 antibody of *C. neoformans* cells which also has low levels of (1-3)-beta-D-glucans and provide further evidence of the effectiveness of RIT in killing the targeted cells.

Our *in vivo* biodistribution results showed a significantly higher accumulation of ^111^In-400-2 antibody in the lungs of the infected mice compared to its accumulation in non-infected ones. This provides a strong evidence of the co-localization of the 400-2 antibody with *B. dermatitidis* cells. There was almost 3 times more ^111^In-400-2 mAb in the lungs of infected mice than non-infected once at 24 h post administration of the antibody (Figure 3). Similar results were observed during the biodistribution of capsular polysaccharide-targeting mAb 18B7 in mice infected intratracheally with *C. neoformans* (Dadachova et al., 2003). The decrease in lung uptake to the background levels in infected mice at 72 h post antibody administration was, most likely, due to the 400-2 mAb coming off (1-3)-beta-D-glucan. However, since the lungs will only experience high radiation doses in the first 4 h after administration of ^213^Bi-400-2 due to ^213^Bi short physical half-life of 46 min, the increased accumulation of the antibody in the lungs early after administration will be sufficient to deliver the cytocidal doses of radiation to *B. dermatitidis* cells.

The biodistribution results provided impetus to pursue RIT experiments. Treatment of infected mice with ^213^Bi-400-2 antibody was able to decrease the lungs CFUs more than 2 logs compared to non-treated infected mice (Figure 4). To the best of our knowledge, this is the first *in vivo* demonstration of the efficacy of RIT targeting fungal pan antigens. This 2 logs (1,000 fold) reduction in infection burden compared favorably with cancer RIT, which typically causes 5–100 fold decrease in the tumor size in comparison with untreated controls in experimental cancer models (Jiao et al., 2019; Quelven et al., 2019). In regard to the *in vivo* specificity of RIT, before performing therapy experiments, we demonstrated that 400-2 antibody was specific for beta-glucan both *in vitro* (cell binding and cell killing results), and *in vivo*, when it concentrated 3 times more in the lungs of the infected mice than non-infected mice during the biodistribution study. If it would be binding non-specifically to Fc receptors on resident macrophages and on other cells in the lungs, there would be no difference in the lungs uptake between infected and healthy mice, and the uptake in the infected lungs would be growing with time, as more inflammatory cells are moving in. ^213^Bi is a powerful alpha-emitter which can cause significant off target effects, including stimulation of immune system. Nevertheless, the effect of ^213^Bi-labeled control antibody on the infection burden was 10 times lower than that of ^213^Bi-400-2. This, in our opinion, is the most direct proof of the specificity of RIT treatment. The effect of the unlabeled 400-2 antibody on the fungal burden should be considered separately. In this regard, FDA approved radiolabeled antibody Zevalin for treatment of non-Hodgkin lymphoma is capable of inducing some ADCC which contributes to the antibody overall efficacy.

In the past we have performed extensive evaluation of RIT toxicity in mouse models of fungal infection using the same radioactive doses of ^213^Bi-labeled antibodies used in the current study. In this regard, the toxicity evaluation in RIT-treated mice infected intratracheally with *C. neoformans* showed the absence of acute hematologic and long-term pulmonary toxicity (Dadachova et al., 2004a,b,c). RIT proved to be much better tolerated by the infected mice than current standard of care Amphotericin B (Bryan et al., 2010). Finally, RIT spared bystander mammalian cells such as macrophages which were able to perform their functions such as nitric oxide production post-RIT exposure (Bryan et al., 2013). The technology of attaching radionuclides to mAbs is mature and can be easily translated from the cancer field into infectious diseases. Importantly, nuclear medicine departments in hospitals world-wide are now routinely treating cancer patients with RIT and are fully equipped for deployment of infectious diseases RIT. Portable imaging equipment is available for imaging patients receiving RIT to ascertain the targeting of radiolabeled mAbs and to monitor the progression of the disease.

In conclusion, our results demonstrate the ability of an anti-(1-3)-beta-D-glucan antibody armed with an alpha-emitter ^213^Bi to selectively kill *B. dermatitidis* cells *in vitro* and *in vivo*. These results provide encouraging first *in vivo* results of the effectiveness of RIT targeting pan-antigens on fungal pathogens. The goal of our group is to pursue a clinical trial of RIT in companion dogs with spontaneous blastomycosis; this research is in progress.

## Data Availability Statement

The datasets generated for this study are available on request to the corresponding author.

## Ethics Statement

The animal study was reviewed and approved by the Animal Research Ethics Board of the University of Saskatchewan.

## Author Contributions

MH, KA, and BD performed the experiment. ED and ES came up with the concept of the study. JN participated in the discussion of the concept and results. MH and ED wrote the manuscript. All authors edited the manuscript.

## Conflict of Interest

The authors declare that the research was conducted in the absence of any commercial or financial relationships that could be construed as a potential conflict of interest.
